# Safety assessment, radioiodination and preclinical evaluation of antinuclear antibody as novel medication for prostate cancer in mouse xenograft model

**DOI:** 10.1038/s41598-023-45984-6

**Published:** 2023-10-31

**Authors:** Thu Minh Chau Nguyen, Lu Duc Chinh Hoang, Thi Khanh Giang Nguyen, Thi Ngoc Nguyen, Quang Chien Nguyen, Thanh Binh Nguyen, Ho Hong Quang Dang, Van Cuong Bui, Thanh Minh Pham, Thi Thu Nguyen

**Affiliations:** 1 Center for Research and Production of Radioisotopes, Nuclear Research Institute, 01 Nguyen Tu Luc Street, Dalat, Lam-Dong Vietnam; 2 Hanoi Medical University, Hanoi, Vietnam; 3 Ho Chi Minh Medicine and Pharmacy University, Ho Chi Minh City, Vietnam; 4 Department of Hematology and Blood Transfusion, Vietnam Military Medical University, Military Hospital 103, Hanoi, Vietnam

**Keywords:** Cancer, Molecular medicine, Oncology

## Abstract

This study aims to provide in vitro and in vivo data to support the utilization of antinuclear antibodies (ANAs) as novel tools for the diagnosis and treatment of prostate cancers. The hematological, biochemical, and histological toxicities of ANAs were assessed at the doses of 5 and 50 μg per mouse. Radiolabeling study was then conducted with ANA and ^131^I using the chloramine T method, and the biodistribution and treatment efficacy were subsequently investigated in a PC3 xenograft model. No changes in clinical behavior or signs of intoxication, necrosis, or malignancy were observed in ANA-treated mice. ^131^I-ANA was obtained in very high yield and radiochemical purity, at 94.97 ± 0.98% and 98.56 ± 0.29%, respectively. They achieved immunoreactivity fraction of 0.841 ± 0.17% with PC-3 cells. Levels of radiolabeled ANAs were 1.15–10.14 times higher in tumor tissues than in other examined organs at 24 h post-injection. The tumor growth inhibition rates were 28.33 ± 5.01% in PC3 xenografts mice treated with ^131^I-ANAs compared with controls and a nearly twofold improvement in median survival was observed. These results demonstrate that radioimmunotherapy of radiolabeled natural ANAs may be an effective treatment for prostate tumors.

## Introduction

We learned that most antibodies that are used for clinical diagnosis and therapy mainly target antigens in the extracellular space and on the surface of tumor cells^1–3^. Scientists have proposed that ANAs, which are found in systemic lupus erythematosus (SLE) patients, may be used therapeutically because of their intracellular mechanism and their ability to enter the nucleus and damage DNA^4–7^. Therefore, exploiting natural ANAs for clinical use has become an attractive approach with great potential, especially when they are conjugated with radioactive isotopes^1,8^.

ANAs are human autoimmune antibodies that are isolated from the serum of SLE patients and include more than 200 different autoantibodies^4^. More than 95% of ANAs and 70% of anti-double-stranded DNA autoantibodies have been reported. As indicated by their name, up to 90% of ANAs bind to nuclear antigens, including DNA and histones, as well as other nuclear antigens, such as Sjögren's syndrome antigen A and B, ribonucleoproteins, junction opener 1, Smith antigen, and scleroderma antigen 70^1,4,9^. Additionally, ANAs also bind to extranuclear antigens, such as U1 ribonucleoproteins, polymyositis, centromere protein B, proliferating cell nuclear antigen, complement 1q, and N-methyl-D-aspartate receptor^1,4,9,10^. On the other hand, high titers of ANAs have been proven to exhibit high sensitivity and specificity^4^, allowing them to enter cells and reach their antigens, especially tumor necrosis areas. Hence, ANAs can be used as therapeutic agents for the treatment of diverse diseases^1,2,5,11^.

To date, many recent studies have consistently shown that people with SLE exhibit a slight increase in the occurrence some malignancies and a decreased risk of certain tumors, such as breast cancer, endometrial cancer, ovarian cancer and prostate cancer^7^. In malignant tumors, neoplastic cells, degenerative cells, dead cells or dying cells are present. Furthermore, in malignant tumors, more than 50% of tumor cell progeny quickly degenerate or even undergo necrosis. Therefore, the membrane permeability of degenerating tumor cells increases, allowing natural antinuclear antibodies to easily bind to nuclear antigens in these abnormal cells^1^.

Relying on this mechanism, many test results have been reported regarding the recombination of monoclonal anti-DNA antibodies, such as the 3E10, 3D8, 5C6, 2C10, H241 and G2-6 antibodies, among which the 3E10 mAb has been tested in patients, and its safety and efficacy have been proven in the clinic^1,7^. Nevertheless, monoclonal anti-DNA antibodies may not represent pathological antibodies in cancer. In addition, such antibodies are expensive, which makes it difficult to use these antibodies to treat patients^12^. However, ANAs are inexpensive, are readily available, have minimal side effects and are effective against a broad spectrum of tumors^4,5^. Many studies on autoantibodies have shown that combination with radioisotopes or chemicals may increase tumor cell sensitivity to these autoantibodies^13^. For example, tumor hypoxia and necrosis can be therapeutically targeted with α-radioconjugates and ^131^I-labeled tumor necrosis therapy (Cotara) in patients with developed lung cancer^14,15^. Therefore, this created an opportunity to promote in vitro and in vivo studies on ANAs and produce new radiopharmaceuticals with potential for use in radioimmunotherapy for treating various tumor types.

**Figure 1:**
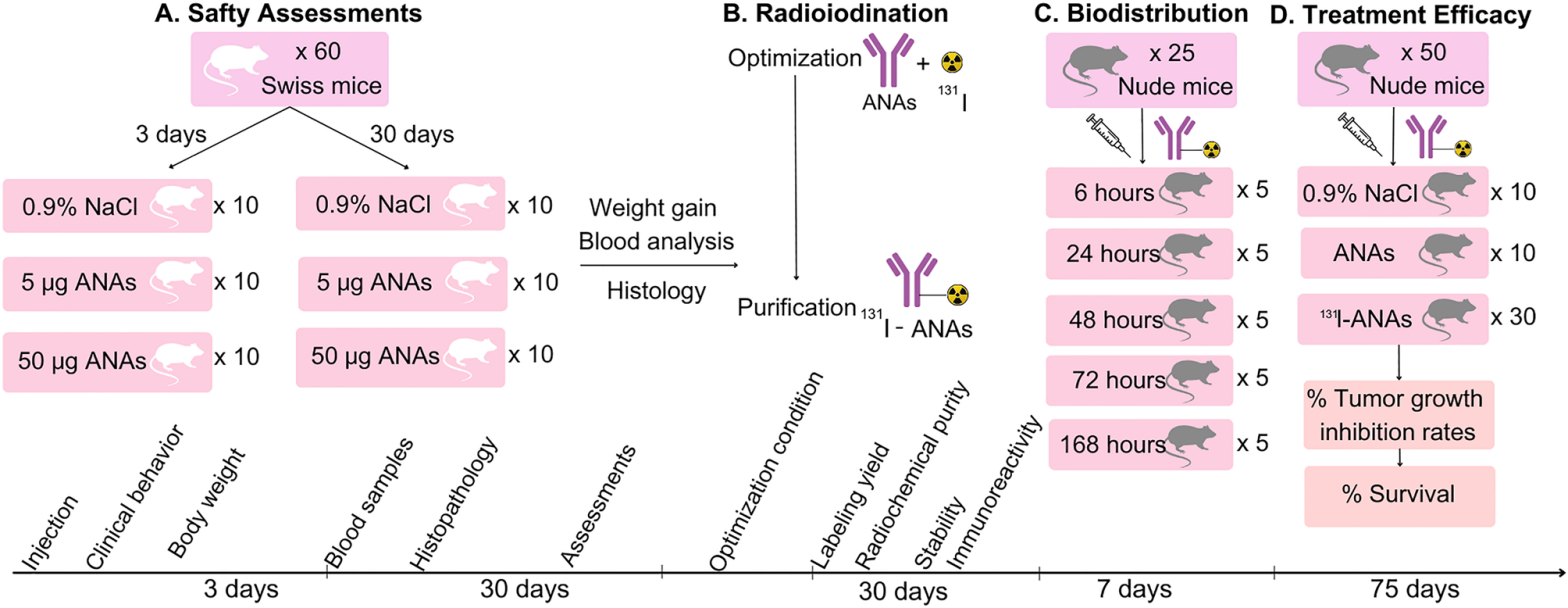
The schematic illustration of study. *ANAs* Anti nuclear antibodies.

Prostate cancer is one of the most prevalent cancers in men, and it has a high mortality rate. According to Globocan 2020, prostate cancer is the second most common cancer, accounting for 14.1% of all new cancer cases and ranking sixth in mortality. The treatment of prostate cancer has become one of the greatest challenges in modern medicine^16^. A novel radioactive therapy called ^68^ Ga/^177^Lu-PSMA is currently being used to treat prostate cancer^17^. Besides, the development of radiopharmaceutical therapy for prostate cancer base on the labeled antibodies such as ^225^Ac-PSMA-617, ^177^Lu/^225^Ac-J591, ^255^Ac/^90^Y-hu5A10, ^177^Lu-SC16, ^225^Ac-YS5 for preclinical application on prostate cancer were evaluated^17^, but it is not currently available in Vietnam because imports are expensive. As a result, a simpler the study's model for prostate cancer treatment was chosen since it was based on ingredients that were easy to obtain and utilize. Similarly, radioisotope ^131^I is a candidate that is suitable for labelling ANAs, and over recent decades, it has been shown to have high clinical safety and gamma 364 keV and beta 606 keV energy^3,18,19^. ^131^I-ANA conjugates have high potential for use in diagnosing and treating cancer. In this report, the safety of ANAs in tissues was tested. Then, we determined the ability of ANAs to be radiolabeled with the ^131^I radioisotope. Finally, the capacity of this labeled conjugate to bind to a prostate cancer cell line, its biodistribution and its efficacy in the treatment of human PC3 tumor-bearing nude mice were evaluated. The schematic illustration of the study was presented in Fig. 1.

## Results

### Safety profile of ANA injection

*Clinical observations:* No mortality was observed in any group. All the mice looked healthy, agile, and ate and drank normally while being fed according to the supplier’s instructions. There were no unusual changes in appearance, behavior or locomotor activities and no clinical signs of toxicity.

*Body weight measurement:* All the mice in Group I gained weight (P < 0.05) on Days 1 and 3, and all the mice in Group II gained weight (P < 0.05) on Days 1 and 30. There were also no significant differences (P > 0.05) in the change in body weight between the treatment groups and control groups (Table 1).

**Table 1:** Body weight measurements and body weight gain (grams) of mice in all groups (n = 10).

Parameters	Group I (3 days)	Group II (30 days)
Baseline weight (g)		
Control	24.94 ± 2.6	25.04 ± 1.76
5 μg ANA	26.64 ± 3.11	24.40 ± 2.26
50 μg ANA	26.03 ± 2.37	24.63 ± 1.63
Weight gain (g)		
Control	3.30 ± 1.36	13.90 ± 2.79
5 μg ANA	2.74 ± 1.24	12.54 ± 4.21
50 μg ANA	2.31 ± 1.01	11.10 ± 3.54

Note that in each group, there were no significant differences in both baseline weight and weight gain between types of treatment (P < 0.05).

*Hematological findings:* In general, the hematological parameters of the 5 μg ANA-injected groups were not significantly different from those of the control groups (P > 0.05). However, an opposite phenomenon was observed in the 50 µg ANA-injected groups, as red blood cell and platelet numbers were consistently lower than those in the control groups (Table 2).

**Table 2:** Count of red blood cells (RBC), white blood cells (WBC), platelets (PLT), serum glutamic-oxaloacetic transaminase (SGOT) and serum glutamic-pyruvic transaminase in mice of all groups (SGPT). n = 9—10 for each group.

Parameters/References^41^	Group I (3 days)	Group II (30 days)
RBC (× 10^12^/L) 7.8–10.6 (× 10^12^/L)		
Control	8.11 ± 0.89	11.69 ± 1.02
5 μg ANA	7.37 ± 0.99	10.20 ± 0.72
50 μg ANA	5.76 ± 0.94*	8.48 ± 0.59*
WBC (× 10^9^/L) 2–10 (× 10^9^/L)		
Control	4.01 ± 2.11	4.28 ± 0.63
5 μg ANA	5.72 ± 2.58	5.66 ± 1.42
50 μg ANA	5.99 ± 1.66	7.11 ± 2.55*
PLT (× 10^3^/L) 900–1600 (× 10^3^/L)^#^		
Control	858.20 ± 242.59	854.60 ± 211.54
5 μg ANA	874.00 ± 482.32	979.40 ± 344.28
50 μg ANA	535.00 ± 233.93*	592.00 ± 106.81*
SGOT (U/L) 54–298 (U/L)^#^		
Control	133.50 ± 73.64	110.80 ± 38.67
5 μg ANA	167.20 ± 137.57	157.60 ± 63.22
50 μg ANA	367.00 ± 264.88*	183.20 ± 89.37
SGPT (U/L) 17–77 U/L^#^		
Control	56.60 ± 41.62	41.00 ± 12.39
5 μg ANA	89.06 ± 64.69	55.80 ± 20.10
50 μg ANA	51.70 ± 23.28	49.10 ± 11.51

(^#^) Mouse biochemistry normal ranges are referred from en.wikivet.net reference. Within a column of the same hematological parameters, those marked with an asterisk (*) are different from the corresponding control group (P < 0.05). Within a column of the same biochemical parameter, those marked with an asterisk (*) are different from the corresponding control group (P < 0.05).

*Biochemical findings:* The injection of ANAs had no significant effect on SGPT (P > 0.05), whereas increases in SGOT were observed in mice that were injected with 50 μg ANAs on Day 3 p.i. (P < 0.05) (Table). However, the level of SGOT returned to the normal range on Day 30 p.i., and this level was not significantly different from that in the control group (P > 0.05) (Table 2).

**Figure 2:**
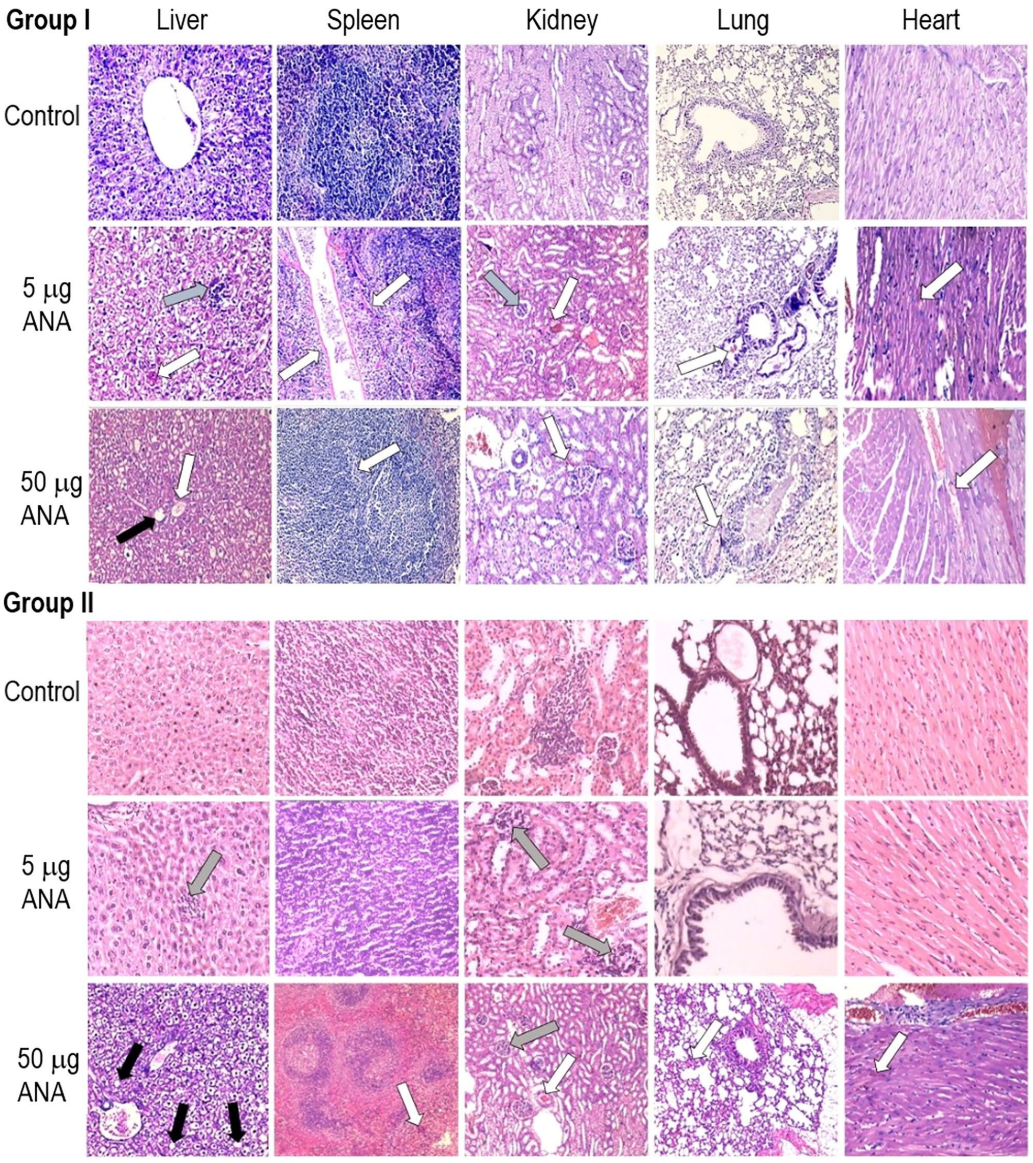
Representative images of different tissues from mice in all groups that were stained with hematoxylin/eosin. No significant abnormalities were evident in any sections. **a** **Group I**, observed on Day 3 after injection; Control group (normal saline); 5 μg ANA-treated group (showed signs of inflammation and congestion, with the liver tissue showing congestion in 2/10 mice and inflammation in 2/10 mice). The spleen and heart exhibited congestion in 2/10 mice, and the lungs and kidneys exhibited congestion in 1/10 mice; 50 μg ANA-treated group (showed signs of congestion in 4/10 mice of each group); **Group II**, observed on Day 30 after injection; Control group (normal saline); 5 μg ANA-treated group (showed signs of inflammation in liver tissue in 1/10 mice); 50 μg ANA-treated group (1/10 mice showed cave-degraded hepatocytes, 1 mouse in each group showed light vein congestion); Ia, IIa: No significant abnormalities were evident in any sections. White arrow: Vein congestion. Gray arrow: Mild reactive inflammation with a cluster of lymphocytes. Black arrow: Cave-degraded hepatocytes.

*Histopathological findings:* Spleen, kidney, heart and lung tissues from every group exhibited no significant abnormalities. No signs of necrosis or malignancy were evident from the microscopic views of these organs. However, noticeable histopathological changes in liver tissues were observed (Fig. 2). In particular, mild reactive inflammation in Group IIb (injected with 5 μg ANAs, Day 30), a few cave-degraded hepatocytes in Group Ic (injected with 50 μg ANAs, Day 3) and additional signs of bile accumulation in Group IIc (injected with 50 μg ANAs, Day 30) were observed, as opposed to the minimal histopathological changes that were in Groups Ia, IIa (normal saline control group) and Ib (injected with 5 μg ANAs, Day 3). There was light venous congestion in the tissues on Day 3, which disappeared on Day 30.

Three days after 5 µg of ANAs were injected, the tissues showed signs of inflammation and congestion, with the liver tissue showing 20% congestion and 20% inflammation. The spleen and heart exhibited 20% congested, although the lungs and kidneys exhibited only 10% congestion. The majority of the tissues returned to their normal functional states during the course of the 30-day follow-up, while the congestion of the livers barely decreased (from 20 to 10%). In addition, congestion and cave-degraded hepatocytes were observed in organ tissues three days after injection. Over 30 days of continued observation, the congestion of liver tissues remained unchanged. However, congestion in other organs decreased.

### Preparation and characterization of ^131^I-ANAs

**Figure 3:**
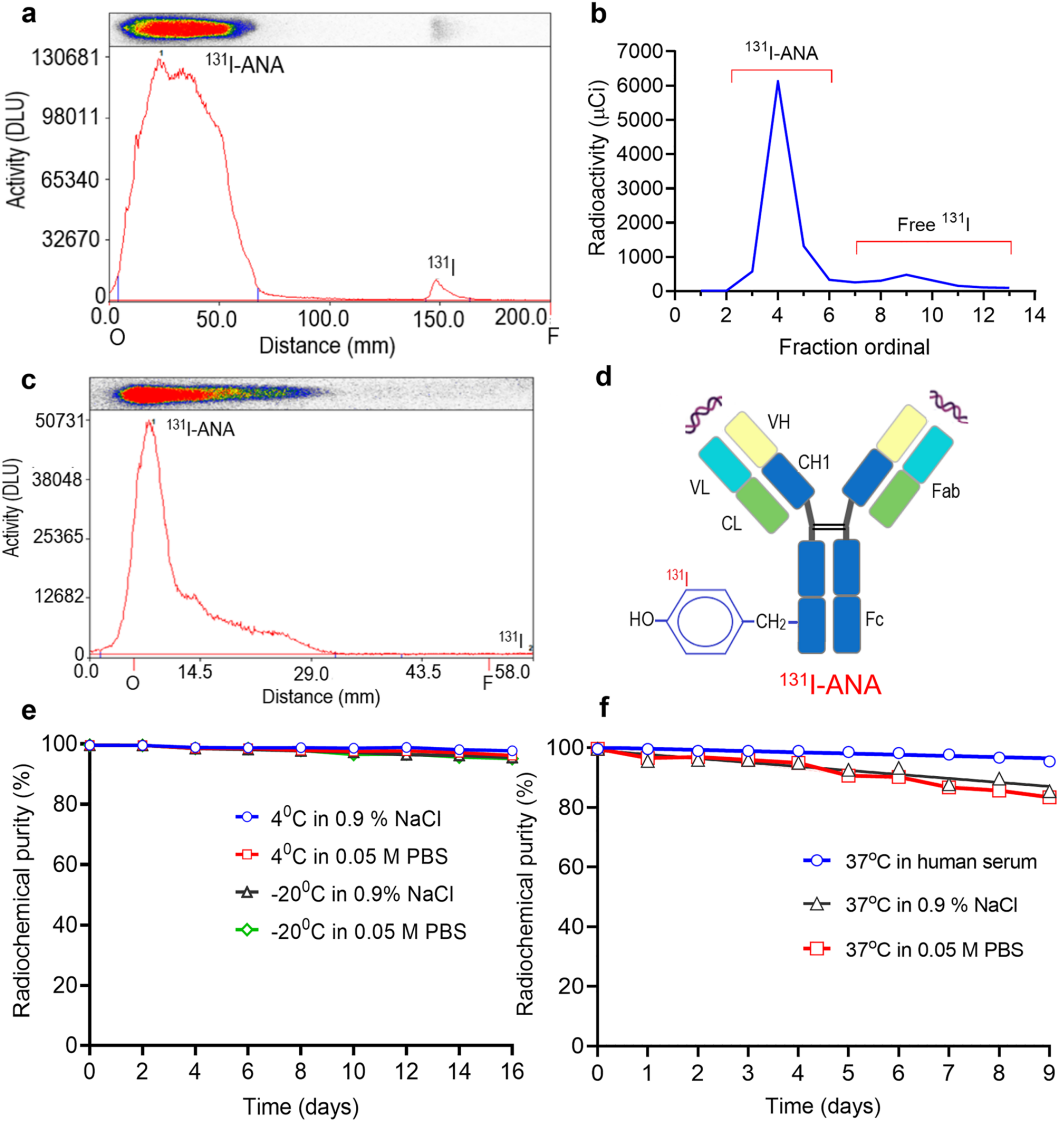
Radiolabeling and quality control of ^131^I-ANAs. (**a **)Efficiency yield of ^131^I-ANAs as determined by paper electrophoresis in 0.025 M phosphate buffer and radioautography (Cyclone, PerkinElmer). Chromatograms were analyzed using OptiQuant 5 software. ^131^I-ANAs stayed at the anode R_f_ = 0.0–0.25, and free ^131^I was separated from the reaction mixtures and migrated to the cathode R_f_ = 0.75–0.85. (**b**) ^131^I-ANAs were collected in fraction no. 3–6 mL after purification through gel sephadex, and free ^131^I was collected in fraction no. 8–10 mL. (**c**) Radiochemical purity of ^131^I-ANAs as determined by Tec-Control-Chromatography (TCC 150–771), Biodex. The strip was placed into a vial containing 0.9% saline, and the strip was developed until the solvent front migrated to the solvent front line. The strip was removed, and then the storage phosphor screen was used. Chromatograms were analyzed using OptiQuant 5 software. ^131^I-ANAs remained at the origin R_f_ = 0.0–0.3, and free ^131^I was separated from the reaction mixtures and migrated to the front R_f_ = 0.9–1.0. (**d**) Hypothetical chemical structures of ^131^I-ANA conjugates,^131^I attachment to the ortho site of the phenol ring tyrosine. (**e**) Stability of ^131^I-ANAs in 0.9% NaCl and 0.05 M PBS after cryopreservation and storage 4 °C. All experiments were performed in duplicate and 3 independent times (n = 3). Simple linear regression analysis of the results was performed by GraphPad Prism Software 8. (**f**) ^131^I-ANAs in 0.05% human serum were stable (96.3 ± 0.31% radiochemical purity) after incubation at 37 °C.

The ANA’s radiolabeling efficiency affected factor included chloramine T, pH, ANA content and incubation time and the results of determination are presented in the Supplementary Fig. S1. Hence, it was chosen in the labeling with ^131^I to generate a ^131^I-ANAs. The yield of radiolabeling was 94.97 ± 0.98% (n = 6), as shown by the representative data in Fig. 3a. The paper electrophoresis results showed a labeled antibody peak with R_f_ ~ 0.1–0.3 at the origin. Under level labeling reaction conditions of 341 ± 33.9 MBq (12.09 ± 1.2 nmol) ^131^I and 1.0 mg (6.6 nmol) ANAs, 1:33 molar ratio of ANAs and chloramine T (chT) and the free ^131^I was removed by gel chromatography, as shown in Fig. 3b, in fractions 9 and 10. The concentration of collected ^131^I-ANAs was 108 ± 11.5 MBq/mL according to a dose calibrator (Capintec ISOMED 2000, USA), pH 7.4. The radiochemical purity was 98.56 ± 0.29% (Fig. 3c). The ^131^I-ANAs in Fig. 3d were collected and had a specific activity of 335.88 ± 36.58 MBq/mg, and the average number of iodine atoms per ANA molecule was estimated to be 1.74 ± 0.19 (Table 3). The radioconjugates were stable after 16 days in 0.9% NaCl in 0.05 M PBS at 4 °C and -20 °C, and they remained stable for 9 days in 0.05% human serum at 37 °C; these samples exhibited 96.3 ± 0.31% radiochemical purity (Fig. 3e and 3f).

**Table 3:** Characteristics of ^131^I-ANA.

^131^I^a^ (nmol)	Ab (nmol)	Efficiency^b^ (%)	RCP^b^ (%)	Conc.^c^ (MBq/μL)	SA^d^ (MBq/mg)	I/Ab^e^ (M/M)	I_a_/Ab^f^
13.12	6.6	94.7	98.58	116.80	361.23	1.99	1.88
11.81	6.6	94.8	98.44	105.23	325.45	1.79	1.70
13.12	6.6	96.4	99.02	118.89	375.45	1.99	1.92
10.89	6.6	95.1	98.15	97.35	304.22	1.65	1.57
10.50	6.6	93.4	98.48	92.15	285.01	1.59	1.49
13.12	6.6	95.4	98.68	117.66	363.90	1.99	1.90
Mean ± SD		94.97 ± 0.98	98.56 ± 0.29	108.01 ± 11.50	335.88 ± 36.58	1.83 ± 0.18	1.74 ± 0.19

Note: Ab, antibody, MBq: Megabecquerel, SA: Specific activity, mole molecular of ANA is about ~ 150 kDa. (^a^) ^131^I (nmol) = used activity of ^131^I (MBq) × 10^3^/222 and divided by 127 (based on ^127^I (stable). (^b^) Labeling efficiency (before purification) and radiochemical purity (after purification) of ^131^I-ANA was determined by paper electrophoresis. (^c^) Concentration = % activity of ^131^I-ANA (MBq) after purification (collected ~ 3.0 mL). (^d^) Specific activity (MBq/mg) of ^131^I-ANA = activity of ^131^I (MBq) × % efficiency/(1 mg ANA × % efficiency after UV). (^e^) I/Ab molar ratio = nmol ^131^I/nmol Ab × (efficiency). (^f^) iodine atoms per Ab molecule.

### Immunoreactivity and saturation binding assay of ^131^I-ANAs

**Figure 4:**
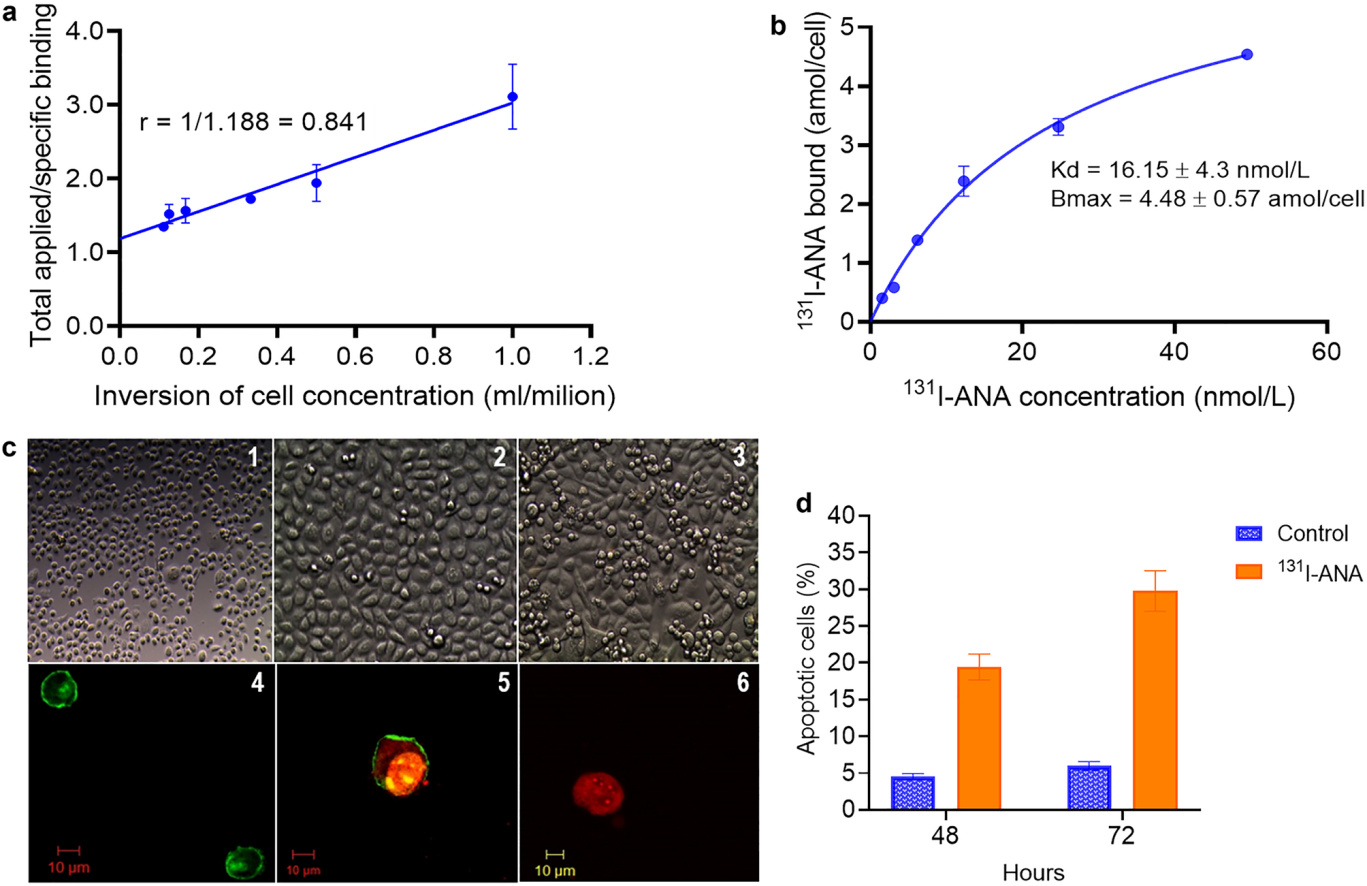
In vitro characterization of ^131^I-ANAs in PC3 cells. (**a**) Immunoreactivity of ^131^I-ANAs against PC3 cells. Duplicates were used to acquire each data point, and the results are shown as the mean ± standard deviation (error bar). Representative results of three independent Lindmo assays to calculate the immunoreactivity (r) of ^131^I-ANAs. By using linear regression analysis 3, immunoreactivity was calculated as the inversion of the Y-intercept. (**b**) ^131^I-ANA saturation binding assay; results are representative of three independent assays of ^131^I-ANA binding to PC3 cells. The Kd and Bmax values are shown as the best-fit value ± standard error. (**c**) PC3 cell line after incubation with ^131^I-ANAs and observed by fluorescence microscopy, (**c**)1: control (after culture), (**c**)2: 48 h, (**c**)3: 72 h, **c**4: PC3 cells stained with annexin V (early stage of apoptosis), (**c**)5: PC3 cells stained with annexin V and PI (late stage of apoptosis), (**c**)6: PC3 cells stained with PI. (**d**) Apoptosis analysis: PBS and ^131^I-ANA treatments at 48 and 72 h. Flow cytometry and FACSDiva Software 6.1.3 were used for investigation and data analysis (n = 3, **P < 0.01; Student’s t test). The results are presented as the mean ± standard error. The apoptotic profile was determined as follows: living cells were Annexin V (−) and PI (−), early apoptotic cells were Annexin V ( +) and PI (−), late apoptotic cells were Annexin V ( +) and PI ( +), and dead cells were Annexin V (−) and PI ( +).

The immunoreactivity fraction (r) of the ^131^I-ANAs was 0.841 ± 0.17% (best fit value ± standard error), which is shown in Fig. 4a. The saturation binding assay revealed a Bmax value of 4.48 ± 0.57 amol/cell (best-fit value ± standard error of the mean), which is approximately 2.7 × 10^6^ atoms/cell. Additionally, the affinity of ^131^I-ANAs for PC3 cells was demonstrated, and the Kd value was 16.15 ± 4.3 nmol/L (best-fit value ± standard error). The nonspecific binding was insignificant (< 3%). The results of the saturation binding assay are shown in Fig. 4b. In the figure, the horizontal axis represents the increasing concentration of ^131^I-ANAs (nmol), and the vertical axis represents the number of antibody binding sites per cell (amol/cell-specific binding). The results were analyzed by nonlinear regression using GraphPad Prism 8 software. When the graph reaches the maximum value, this indicates saturation. The nonspecific binding fraction was less than 3%, and the difference was significant (P < 0.05%).

### Cell apoptosis analysis

The percentage of apoptotic cells in the ^131^I-ANA-treated groups was significantly higher than that in the control groups (P < 0.01). After 48 h of the experiment, early apoptosis was induced in 19.43 ± 2.96% of PC3 cells that were treated with ^131^I-ANAs, and this proportion was 4.28 times higher than that in the control group (4.53 ± 1.1%). At 72 h, ^131^I-ANAs caused 29.77 ± 4.95% of PC3 cells to undergo early apoptosis, increasing the proportion of apoptotic cells by approximately 5 times compared with that in the control group (6.0 ± 1.2%). (Fig. 4c and 4d).

### Pharmacokinetics, biodistribution and treatment efficacy of ^131^I-ANAs

**Figure 5:**
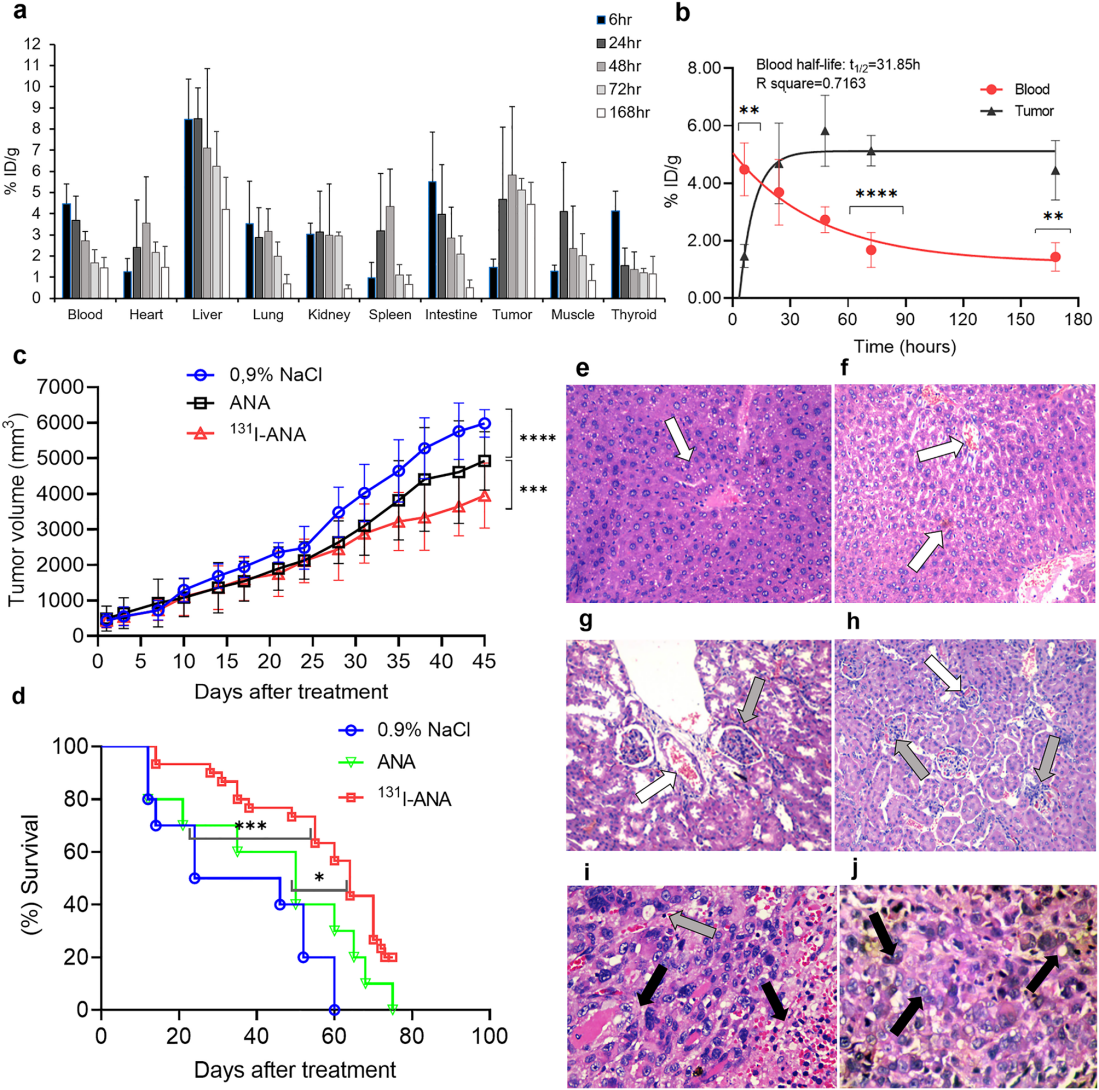
Analysis of ^131^I-ANA biodistribution in vivo and inhibition of tumor growth (**a**) Biodistribution of ^131^I-ANAs in tissue of mice with PC3 xenografts (n = 5). The results are presented as the mean ± SD (error bars). (**b**) Blood clearance and tumor concentration of ^131^I-ANAs with time. (**c**) Tumor growth in nude mice bearing PC3 tumor xenografts; mean ± SD, n = 10 in the 0.9% NaCl and ANA groups, n = 30 in the ^131^I-ANA group (****P < 0.0001, ^131^I-ANAs vs. 0.9% NaCl; ***P < 0.001, ^131^I-ANAs vs. ANA, two-way ANOVA with Tukey's multiple comparisons test. The tumor growth inhibition rates were expressed as the mean and SE (13.21 ± 6.79%, ^131^I-ANAs vs. ANA; 28.33 ± 5.01%, ^131^I-ANAs vs. 0,9% NaCl and 17.41 ± 5.18%, ANAs vs. 0,9% NaCl) (**d**) Kaplan‒Meier survival curve showing the effects of ^131^I-ANAs on the median survival of PC3 tumor-bearing mice (***P = 0.0006; GraphPad Prim 8; log-rank (Mantel‒Cox) test; chi-square 14.89, df = 2). Compared with 0.9% NaCl (control), treatment with ^131^I-ANAs or ANAs resulted in a 1.83- (64 vs. 35 days) or 1.43-fold (50 vs. 35 days) increase in median survival, respectively (GraphPad 8, survival). (**e**), (**f**) Light micrograph of HE staining (200 ×) in liver, which showed hemorrhagic zones and mild degeneration (arrow white) at 72 and 168 h after injection. (**g**), (**h**) Light micrograph of HE staining (200 ×) of kidney, which showed replicated endothelial cells of the artery (gray white) at 72 and 168 h after injection. (**i**), (**j**) Light micrograph of HE staining (400 ×) of the tumor, which showed large nucleoli, bizarre nucleoli, and abnormal mitotic nuclei (black arrow) at 72 and 168 h after injection. Notes: White arrow: hemorrhagic zones; Gray arrow: reactive inflammation; Black arrow: large nucleoli, bizarre nuclei, abnormal mitotic nuclei.

The biodistribution of ^131^I-ANAs at 6, 24, 48, 72, and 168 h p.i. is shown in Table 4 and Fig. 5a. In the first 48 h p.i., ^131^I-ANAs were generally distributed to the PC3 tumor as much as to other organs, with marginal differences at 24 h and 48 h p.i. (P < 0.05). Then, ^131^I-ANAs were predominantly retained in tumor tissues 72 h after administration (P < 0.001), with the only exception being liver tissue. Although the uptake of ^131^I-ANAs by the liver was even higher than that by the tumor in the first 72 h p.i., the levels gradually decreased from their peak of 8.47 ± 1.88% ID/g at 6 h p.i. to 4.22 ± 1.5% ID/g at 168 h p.i.. Moreover, ^131^I-ANAs accumulated and remained stable at the PC3 tumor site, with a fluctuation within 1.38% ID/g from its highest value of 5.83 ± 3.24% ID/g at 48 h p.i. Other noncancerous organs also exhibited a pattern of decreasing uptake after the levels peaked in the first 24 h, but these other organs exhibited faster rates of decline than the liver. The levels of ^131^I-ANAs in tumor tissues were 1.15 to 10.14 times higher than those in other examined organs 24 h p.i., except for the levels in liver tissues. The half-life of labeled ANAs in the blood was 31.85 h (R square = 0.7163, Fig. 5b). The body weight examination was exhibited no significant differences compared to mice treated with 0.9% NaCl, ANAs and ^131^I-ANAs (P > 0.05) which are shown in Supplementary information, Fig. S2. The initial tumor sizes in the 0.9% NaCl, ANAs and labeled ANAs treatment groups were 441 ± 192, 495 ± 254 and 443 ± 211 mm^3^, respectively. After 45 days, the tumor sizes were 5982 ± 384, 4925 ± 826 and 3949 ± 917 mm^3^, respectively. Mice that were treated with ANAs or ^131^I-ANAs had significantly smaller tumor volumes than control mice (****P < 0.0001; two-way ANOVA with multiple comparisons), and mice that were treated with ANAs had significantly smaller tumor volumes than ^131^I-ANA-treated mice (***P = 0.0006; two-way ANOVA with multiple comparisons). The tumor growth inhibition rates of the ^131^I-ANA and ANA groups compared with the 0.9% NaCl group were 28.33 ± 5.01% and 17.41 ± 5.18%, respectively (Fig. 5c). The tumor growth inhibition rates of the ^131^I-ANA compared with the ANA group were 13.21 ± 6.79%. Kaplan‒Meier survival analysis showed that treatment with ^131^I-ANAs or ANAs increased the median survival of mice with PC3 xenografts by 1.83-fold or 1.43-fold, respectively, compared with treatment with 0.9% NaCl (P = 0.0001; chi-square 14.44; or P = 0.209; chi-square 1.57, log-rank (Mantel‒Cox) test) (Fig. 5d). The survival of mice was also monitored until 50% of the control group had died. On Day 45, the mortality rates in the groups that were treated with ^131^I-ANAs, ANAs and 0.9% NaCl were 20, 40 and 50%, respectively. Interestingly, 100% of mice in the 0.9% NaCl and ANA groups had died by Day 60 and Day 75, while 20% of the mice that were treated with ^131^I-ANAs survived. Histopathological analysis of liver and kidney tissues showed hemorrhagic zones, mild degeneration, and dilated portal vein disorder, but no human prostate carcinoma cell metastasis (Fig. 5e-h). Tumor tissues with necrotic or hemorrhagic areas, irregular nuclei, large nucleoli, and bizarre nuclei are visible in Fig. 5i and j.

**Table 4:** Biodistribution of ^131^I-ANA in different tissues of PC3 xenograft mice, N = 5 per condition.

Organ/tissue	% ID/g				
	6 h	24 h	48 h	72 h	168 h
Blood	4.48 ± 0.92**	3.69 ± 1.14	2.73 ± 0.45	1.68 ± 0.60****	1.44 ± 0.50**
Heart	1.25 ± 0.63	2.41 ± 2.23	3.57 ± 2.19	2.16 ± 0.52***	1.46 ± 0.99*
Liver	8.47 ± 1.88**	8.50 ± 1.45	7.11 ± 3.74	6.23 ± 1.64	4.22 ± 1.50
Lung	3.53 ± 2.01	2.88 ± 1.41	3.17 ± 1.07	2 ± 0.67***	0.67 ± 0.44**
Kidney	3.04 ± 0.51**	3.13 ± 1.93	2.98 ± 2.44	2.95 ± 0.19**	0.44 ± 0.20**
Spleen	0.98 ± 0.71	3.2 ± 2.71	4.34 ± 1.77	1.11 ± 0.48****	0.65 ± 0.46**
Intestine	5.51 ± 2.35	3.96 ± 2.36	2.86 ± 1.46	2.08 ± 0.88 **	0.51 ± 0.37**
Tumor	1.47 ± 0.40	4.69 ± 3.40	5.83 ± 3.24	5.13 ± 0.53	4.45 ± 1.03
Muscle	1.28 ± 0.30	4.09 ± 2.34	2.36 ± 0.20	2.01 ± 1.05**	0.84 ± 0.75**
Thyroid	4.14 ± 0.94*	1.56 ± 0.83	1.38 ± 0.81	1.20 ± 0.21***	1.17 ± 0.83**
T/B	0.33	1.27	2.14	3.05	3.10
T/L	0.17	0.55	0.82	0.82	1.06
T/K	0.48	1.50	1.96	1.74	10.14
T/M	1.15	1.15	2.47	2.55	5.32
T/Th	0.35	3.01	4.24	4.27	3.81

Within the same column, asterisk (*) means that at the same time point post-injection, the levels of ^131^I-ANA in organs differ significantly from those in the tumors (*P < 0.05, **P < 0.01, ***P < 0.001, ****P < 0.0001, one-way ANOVA tests with Dunnett's T3 multiple comparisons). Test details on 168 h: Tumor with blood (p = 0.076, t = 5.9) Tumor with heart (p = 0.012, t = 4.7), Tumor with lung (p = 0.0043, t = 7.5), Tumor with kidney (p = 0.006, t = 8.5), Tumor with intestine (p = 0.0031, t = 8.1). T/B, tumor to blood ratio; T/M, tumor to muscle ratio; T/L, tumor to liver ratio; T/K, tumor to kidney ratio; T/Th, tumor to thyroid ratio.

## Discussion

In this study, we demonstrate that human ANAs could be labeled with ^131^I with high efficiency. The optimal research conditions led to ^131^I-ANA conjugation with a high labelling efficiency of 94.97 ± 0.98%, which is consistent with published studies. Furthermore, the successful binding of the ANAs to the tumor cell line indicates its potential for use in clinical applications. In addition, we also determined, including by histopathology, that concentrations of ANAs between 5 and 50 µg are safe in experimental mice. The first reason we chose ANAs in this study is because natural ANAs exists inside the body, even in elderly individuals, which proves that ANAs are safe for living creatures. Second, ANAs can destroy cancer cells in the necrotic area^1,5,8^. Furthermore, several authors have shown that SLE patients are protected against several cancers, such as lung, prostate, and breast cancer^7,23^. Recently, two-thirds of diseases have been shown to be related to targets within nucleus, especially dsDNA and nucleosomes^1,4^. In addition, ANAs are easily isolated from SLE patients and are used as a therapeutic agent^1,4,5^. Regarding a hypothetical mechanism, ANAs bind to nucleosomes that are released from apoptotic tumor cells, circulate inside and outside the tumor and kill tumors via antitumor mechanisms, such as ADCC/immune effector cells^5^. These mechanisms have enabled the development of novel methods for the diagnosis and treatment of several cancers, as proposed in previous studies^30^. In terms to the ANA dose that was used in the experimental mice, we administered between 5 µg and 50 µg ANAs per mouse, and this dose was based on a previous similar study that used humanized monoclonal antibodies, such as rituximab, cetuximab, and nimotuzumab. The safety results showed the weight, hematological parameters and biochemical parameters of the mice that were treated with 5–50 µg ANA were within normal ranges. Studies have reported that the dose for Bexxar antibody treatment is 450 mg per patient, that for rituximab treatment is 225 mg per patient^31^, and that for nimotuzumab treatment is 200–400 mg per patient (average 2.8–8.5 mg/kg); nevertheless, the dose for treatment with radiolabeled antibodies was approximately 0.36–1.0 mg/kg. Therefore, a dose of 1 mg/kg was chosen for use in the experiments^32,33^. When injecting ANA, an antibody highly presented in SLE patients, into healthy individuals, concerns about recreating SLE symptoms may be raised. Although ANAs present at high titer in SLE patients, they can also be found in healthy people, at low ANA titer (< 1:160)^34^, and the understanding of autoimmune physiology revealed that auto-antibody does not warrant the acquisition of SLE. However, ANA titer seems to be related to disease severity: if the ANA titer is high (e.g. 1:640, 1:1280, or 1:2560), this indicates a more severe disease. If the ANA titer is low (e.g. 1:40, 1:80, or even 1:160), there is often no autoimmune disease^4^. Different tissues from the mice in the different groups showed no significant abnormalities, with only mild reactive inflammation in the liver and kidney. The injected ANAs did not result in the destruction of normal tissues. However, additional studies are required to determine the safety of ^131^I-ANAs with respect to other tissues.

We demonstrated that ANAs can be labeled with the radioisotope ^131^I, which enhanced in in vitro and in vivo ANA studies. Additionally, ^131^I was selected because it is suitable for targeted radioimmunotherapy in the treatment of small tumors, such as prostate cancer, and treatment can be followed with SPECT imaging. Second, ^131^I emits both γ- and β-rays. Furthermore, ^131^I is a well-known radioisotope and is frequently used for treating thyroid cancer. We used a traditional radiolabeling modality that is similar to that used for labeling antibodies with ^131^I, such as ^131^I-tositumomab, ^131^I-rituximab, ^131^I-nimotuzumab, ^131^I-omotuzumab and ^131^I-bevacizumab; the results showed high labelling efficiency (> 94%), similar to a previous study that reported > 91% efficiency, high radiochemical purity (> 98%) and in vitro stability (> 96%) for 16 days^22,23,26^. The immunoreactivity study results showed that radiolabeled ANAs did not significantly affect immunoreactivity or affinity. The immunoreactivity fraction r of ^131^I-ANA was 0.841 ± 0.17%, which was similar to previous reports on radioimmunoconjugates that reported ~ 0.8 for ^31^I-nimotuzumab, > 80% for ^131^I-bevacizumab^26,35^ or 83% for ^131^I-omburtamab (8H9)^36^. The binding capacity of the ^131^I-ANA complex to PC3 cancer cells was measured; the K_d_ value was approximately 16.15 ± 4.3 nmol/L (10^–9^ M), and the number of sites on the cytoplasmic antigen calculated from the B_max_ value was 2.7 × 10^6^. The 10^–9^ M data confirm antibody binding affinity after radiolabeling^3^. These results are similar to those reported by studies on ^177^Lu-nimotuzumab (K_d_ values were 28.6 ± 3.9 nmol/L and 32.1 ± 5.5 nmol/L, and binding sites per cell were 1.7 × 10^6^ and 7.1 × 10^5^ for A431 and SNU-C2B cells, respectively^36^), ^131^I-nimotuzumab (Kd value ~ 24.7 nmol/L and 19.2 × 10^5^ binding sites per cell with Hep-2 cells)^22^, and ^99m^Tc-fanolesomab (K_d_ ~ 1.6 × 10^–11^ M and 5.1 × 10^5^ binding sites per human neutrophil^37,38^). The results showed that the specificity and affinity of human ANAs for tumor nuclear antigens were higher than or equivalent to those of specific monoclonal antibodies on the tumor cell membrane. After incubating PC3 cells with ^131^I-ANAs for 48 h and 72 h, the PC3 cells were observed, and the results revealed an effect of ^131^I-ANAs on inhibiting PC3 cell growth. These results are similar to results from studies on the tumor necrosis-inducing effects of the ^131^I-factor-related apoptosis-inducing ligand (TRAIL-I-131) to the A549 and H358 cells^39^. Consequently, the data related to the ^131^I-ANA biodistribution in PC3 tumor-bearing mice indicated high radioactivity concentrations in the liver, kidney, blood, and lungs and retention in the heart and blood after 168 h of monitoring. It would be reasonable that the biological half-life of ANAs was 3–4 weeks. After one week of follow-up, the radioactivity concentrations in cancer tissues were 10 times higher than those in kidney tissues, 5 times higher than those in muscle tissues, and 3 times higher in blood. Perhaps one of the reasons was the increased levels of DNA in the necrotic regions of the tumor, indicating the possible use of ^131^I-ANAs in therapeutic trials and follow-up scintigraphy. The results were relatively similar to those from other similar studies on the biodistribution of radiolabeled antibodies in xenografts. Additionally, these findings were consistent with the 20 μg ^64^Cu-cetuximab per mouse in a study by Yukie et al.^40^. We demonstrated that labeled ANAs exert antitumor effects in PC3 tumors with a large sample size (n = 30 mice) and repeated injection. The in vivo data on the tumor growth inhibitor rate and survival were significant, and ^131^I-ANA injection resulted in a 1.83-fold increase in the median survival of mice compared to the control.

## Conclusions

In summary, we have shown that natural antibodies isolated from SLE patients are safe to utilize in experimental mouse models. Concurrently, ANAs were successfully radiolabeled with ^131^I via high-efficiency radioiodinated conjugation, and these labeled antibodies showed high radiochemical purity as well as stability in human serum. The remarkable results of in vitro and in vivo ^131^I-ANA characterization in prostate cancer cell lines and PC3 xenograft mouse models demonstrated that this radioimmunoconjugate is a potential agent that could be suitable for further therapeutic study for treating not only prostate cancer but also various other types of cancer. Further studies should concentrate on the dose so that radioconjugated ANAs can be used clinically and explored in other solid tumor models to exploit their therapeutic potential.

## Methods

### Ethics declarations

The study is reported in accordance with ARRIVE guidelines. All the animal care and experimental protocols were approved by the animal ethics committee of the Vietnam Military Medical University and followed the guidelines of the Animals (072/13). Throughout the experimental period, the mice were raised in a clean room and provided with filtered air, food and drink according to the manual of the mouse provider.

### ANA safety studies

Sixty 8-week-old male Swiss mice were obtained from Nha Trang Pasteur Institute (Nha Trang, Vietnam), randomly divided into 2 groups of equal size, and investigated for 3 or 30 days. In each group, the mice were divided into 3 subgroups of 10 mice each. The mice in subgroup a were injected with normal saline and served as control groups, while 5 μg or 50 μg ANAs (Probactive Biotech, Inc. CA, USA)^8^ were injected into the mice in subgroups. The clinical behavior of all the mice was observed twice daily. For the mice in Group I, body weight was measured on Days 1 and 3 postinjection (p.i.). Blood samples were harvested on Day 3 to count RBCs, WBCs, and PLTs using a hematological analyzer (XN-1000, Sysmex, Japan), and quantification of SGOT and SGPT was performed using a biochemistry analyzer (AU 680, Beckman Coulter, Japan). Similar investigations were performed on the mice in Group II, but the timing of body weight measurement (on Days 1 and 30) and blood sample analysis (on Day 30) were altered. At the end of the experimental period, the mice were euthanized with ketamine and xylazine. Their spleens, kidneys, hearts, lungs and livers were excised, fixed in 10% neutral buffered formalin for 24 h before undergoing a series of histopathological processes for sample preparation^20^, hematoxylin and eosin staining^21^ and finally examination by light microscopy. To compare the data from repeated weight measurements, a paired t test was utilized. To compare data between 2 groups, Student’s t test with Welch’s correction or the Mann‒Whitney U test for nonparametric data was utilized. To compare data among multiple (> 2) groups, one-way Welch’s ANOVA test followed by Dunnett’s post hoc test for comparison to a specific group of interest was utilized.

### Radioiodination of ANAs and quality control

Radiolabeling of ANAs (1.65 mg/mL, in phosphate-buffered saline (PBS), pH 7.4) with sodium iodine-^131^I (Na^131^I, 222 GBq/mg, 7.4 GBq/mL in 0.05 N NaOH, > 99.9% radionuclide purity, Dalat, Vietnam) was performed with the Chloramine T method^22,23^ base on results on reaction optimization. Briefly, 100 μL of 0.5 M PBS, pH 7.4, 606 μL (1.0 mg) of ANAs, 50 μL (296–370 MBq) of Na^131^I and 50 μL (50 μg) of Chloramine T (Sigma) were consecutively added to the reaction vial, lightly mixed and incubated for 5 min at room temperature. To stop the reaction, 75 μL (150 μg) of sodium metabisulfite was added and gently mixed. The process of labeled ANA purification was performed on a gel filtration pD10 column (Sephadex G25, GE Healthcare Buckinghamshire, UK), and the samples were preequilibrated and eluted with 0.05 M PBS, pH 7.4, supplemented with 1.0% human serum (Sigma‒Aldrich). Fractions 4, 5 and 6 exhibited the highest radioactivity, and they were pooled and then subjected to sterile filtration with a 0.2 µm filter (Sartorius, Goettingen, Germany). The specific activity of ^131^I-ANA in MBq/mg was estimated by calculating the radioactivity of ^131^I and the mass of ANAs after UV spectrophotometry at 280 nm. The number of ^131^I atoms that bound to each ANA molecule was calculated by multiplying the molar ratio of iodine per molecule of ANA by the labeling efficacy as previously described^22,23^. In the experiment, we used 1.0 mg (6.6 nM) of ANA and 370 MBq (13.12 nM) of ^131^I based on the specific activity of ^131^I, which is 222 GBq/mg, and the molecular weight of the ANAs, which is 150 kDa. The labeling efficacy and radiochemical purity were determined by paper electrophoresis (0.025 M PBS, pH 7.5, 300 V, 60 min, Whatman 1 paper strips)^24^ and confirmed by Tec-Control Chromatography strips (Biodex Medical Systems, Inc., NY)^25^. After iodination, aliquots of ^131^I-ANA were stored in 0.9% NaCl in 0.05 M PBS at either 4 °C or – 20 °C for 16 days (9.25 MBq, 13.7 μg ANA, 0.5 μL), and in human serum and 0.9% NaCl in 0.05 M PBS at 37 °C for 9 days (12.95 MBq, 19.2 μg, 0.5 μL) for stability studies using paper electrophoresis. The radioactivity in the paper electrophoresis strips was scanned by radioautography (Perkin Elmer Life Science)^26^. The preparation was performed in 6 batches during the experiment.

### Immunoreactivity

PC3 cells (ATCC CRL-1435) were cultured in F-12 K medium (ATCC, Maryland) supplemented with 10% fetal bovine serum (Gibco) and 1% penicillin/streptomycin (Life Technologies, CA) in a 5% CO_2_ atmosphere at 37 °C until they reached approximately 80% confluence. Determination of ^131^I-ANA immunoreactivity was performed based on Lindmo’s cell-binding assays^27^. In short, a fixed concentration of ^131^I-ANAs (25 ng/mL (20.3 KBq/mL) were incubated for 2 h at 37 °C with increasing concentrations of PC3 cells (0.2 to 9 million cells/mL). To determine nonspecific binding, 100-fold excess cold nonradiolabeled ANAs were used to saturate specific binding sites before adding ^131^I-ANA. After incubation, the radioactivity in the tubes was measured, the cells were transferred to the wells in a Millipore tray (Filter Plate 96-Well, Millipore), the Millipore filter system was installed, the pump was turned on to drain the solution. Then, the cells were washed with phosphate buffer 4 times, the membrane filter containing cells was collected, and the radioactivity was measured with a gamma counter (Caprac 13, Capintec) together with that of the total tube. A double inverse plot of total applied/specific binding activities (after nonspecific binding activities were subtracted) as a function of 1/cell concentration yielded a straight line.

### Saturation binding assay

A fixed number of PC3 cells (2.0 × 10^5^ cells) were incubated with or without 100-fold excess cold nonradiolabeled ANAs to block nonspecific binding; then, the cells were incubated for 3 h at 37 °C with a doubling concentration of ^131^I-ANA (1 nM to 50 nM)^28^. Then, the cells were washed and collected through a Millipore membrane, and radioactivity was measured. The binding affinity (Kd) of the ^131^I-ANAs and the maximum binding capacity (Bmax) were determined by plotting the concentration of ^131^I-ANAs bound to the cells (amol/cell) as a function of the ^131^I-ANA concentration and using nonlinear regression analysis.

### Cell apoptosis analysis

PC3 cells were subcultured into 4 groups that each contained three 25 cm^2^ culture flasks (Corning) with 10^5^ cells each. Two groups were continuously cultured in F12K medium with the addition of 10.3 μg (100 μCi/118 μL) of ^131^I-ANA, while the other 2 groups were treated with PBS as a control. After 48 h and 72 h, the apoptosis-inducing property of ^131^I-ANAs was determined by using an Annexin V-PI dead cell apoptosis kit (Invitrogen, Massachusetts, USA) according to the manufacturer’s instructions. Briefly, 100 μL of a 10^6^ cell/mL suspension was incubated in the dark with 100 μL of Annexin V/PI for 15 min at room temperature. The cells were observed by fluorescence microscopy. Flow cytometry (Beckman Coulter, CA) and FACSDiva Software 6.1.3 were used for investigation and data analysis.

### Evaluation of biodistribution and treatment efficacy

To establish tumor-bearing mouse models, 10^8^ PC3 cells (100 μL) were subcutaneously injected into the right thigh of 8-week-old BALB/c nude mice (Charles River Laboratory, Morocco, USA). The tumor volumes of 75 mice reached ~ 400 mm^3^, which was calculated with the tumor length (L) and width (W) according to the following formula: LW2 × 0.5^22^. In the biodistribution experiment, 25 BALB/c mice with PC3 xenografts were divided into 5 groups of 5 mice each to perform an investigate with 5 time points (6, 24, 48, 72, and 168 h p.i.). ^131^I-ANAs (15.3 μg; ~ 5.18 MBq/100 μL) were injected into the mice via the tail vein. At each designated time point, the mice in the corresponding group were sacrificed, and their organs of interest and blood samples were collected, weighed, cut and examined to measure residual radioactivity. By comparing the radioactivity of the injected dose (ID) and considering the natural disintegration of ^131^I, the biodistribution of ^131^I-ANAs was expressed as the percentage of ID per gram of tissue at the time of measurement (% ID/g)^29^. The blood clearance half-life of the radio-conjugates was calculated using one-phase exponential decay (GraphPad Software Inc.). In the treatment experiment, 50 BALB/c mice with PC3 xenografts were divided into 3 groups and given six doses of weekly injections with 100 μl 0.9% NaCl (n = 10), 20 μg ANAs (n = 10) or ~ 7.33 MBq/20 μg ^131^I-ANAs (n = 30). In the ^131^I-ANAs group, Lugol was added to the drinking water 2 days before the treatment began and refreshed daily, to block the thyroid from taking up free radioiodine. The tumor size and body weight were measured every 3–4 days. The tumor growth inhibition rate (%) was calculated as follows: 100 − (the mean tumor size of the treatment group/mean tumor size of the control group) × 100^22^. The treatment efficacy in the mouse groups was analyzed using GraphPad Prism 8. Histological examination of the tumor, liver and kidney samples from the 72 and 168 h groups was carried out with hematoxylin and eosin staining and observation under light microscopy.

### Statistical analyses

Data are expressed as the means ± SDs/SEM. Statistical analysis was performed with GraphPad Prism 8, and a P value of < 0.05 was considered significant. The normality of the datasets was determined by the Shapiro‒Wilk test. Student’s t test was used in this research to analyze and estimate the differences in body weight, hematological and biochemical parameters and biodistribution. Alternatively, the Kruskal‒Wallis test followed by Dunn’s post hoc test was used to analyze nonparametric data.

### Supplementary Information

Supplementary Information 1.

## Data Availability

All data generated or analyzed during this study are available from the corresponding author on reasonable request.
