# Supplementary material for: Safety assessment, radioiodination and preclinical evaluation of antinuclear antibody as novel medication for prostate cancer in mouse xenograft model
Source: Sci Rep. 2023 Oct 31;13:18753. doi: 10.1038/s41598-023-45984-6 (PMC10618443; doi:10.1038/s41598-023-45984-6)
Supplement: Supplementary file 1 — Supplementary Information 1. [file 41598_2023_45984_MOESM1_ESM.docx]

**SUPPLEMENTARY INFORMATION FOR**

**SAFETY ASSESSMENT, RADIOIODINATION AND PRECLINICAL EVALUATION OF ANTINUCLEAR ANTIBODY AS NOVEL MEDICATION FOR PROSTATE CANCER IN MOUSE XENOGRAFT MODEL**

Thu-Minh-Chau Nguyen^2^*^#^*, Lu-Duc-Chinh Hoang^3#^, Quang-Chien Nguyen^4^, Thi-Khanh-Giang Nguyen^1^, Thi-Ngoc Nguyen^1^, Thanh-Binh Nguyen^1^, Ho-Hong-Quang Dang^1^, Van-Cuong Bui^1^, Thanh-Minh Pham^1^, Thi-Thu Nguyen^1*^*^#^*,

*^1^Center for Research and Production of Radioisotopes, Nuclear Research Institute, Dalat, Vietnam;*

*^2^Hanoi Medical University, Vietnam;*

*^3^Ho Chi Minh Medicine and Pharmacy University, Vietnam;*

*^4^Vietnam Military Medical University, Hanoi, Vietnam;*

*^*#^Corresponding author*

Thi-Thu Nguyen, Ph. D

Center for Research and Production of Radioisotopes, Nuclear Research Institute

01 Nguyen Tu Luc Street, Dalat city, Lam-dong province, Vietnam

Tel.: 84-918223739, Fax: 84-263-3821107, E-mail address: [ngthithu2014@gmail.com](mailto:ngthithu2014@gmail.com)

*^#^Co-first authors*

**Supplementary information:**

**1) Supplementary method S1:** Factors affecting labeling efficiency including chloramin T, pH, ANA content and incubation time were investigated to get the optimum radiolabeling condition.

**Supplementary results S1:** The results of ^131^I-ANAs conjugation formation are presented in the following **Fig. S1**.

Results of effect of chloramine T content: At room temperature, pH 7.4, 1 mCi ^131^I, 100 μg ANA and chloramine T amount of 1, 2, 5, 10, 20, 50 μg, the labeling yield (in percentage) were 89.42 ± 1.41 , 92.61 ± 1.07, 96.52 ± 1.35, 96.74 ± 1.11, 95.67 ± 2.21, 94.42 ± 1.08, respectively.

Results of effect of pH: At pH value of 3, 4, 5, 6, 7, 8 and 9, the labeling yield (in percentage) were 90.34 ± 2.54, 91.23 ± 1.07, 95.16 ± 1.05, 95.32 ± 1.06, 95.23 ± 1.07, 95.25 ± 1.10, 93.58 ± 0.90, respectively.

Results of effect of ANA content and ^131^I: At ^131^I of 1 mCi, with 2, 10, 20, 50, 100, 200 μg ANA, which correspond to molar ratios (moles of I/mole of ANA) 130.0, 26.0, 13.0, 5.2, 2.6 and 1.3, Radiolabelling yield (in percentage) was determined to be 61.34 ± 2.03, 95.21 ± 0.90, 95.22 ± 1.01, 95.42 ± 1.03, 95.13 ± 1.16, 95.13 ± 0.88, respectively.

Results of effect of incubation time on labeling: At room temperature, the labeling efficiencies (in percentage) of ^131^I-ANA after 1, 5, 10, 20, 30 and 60 min of incubation were 95.18 ± 1.88, 95.23 ± 1.15, 95.54 ± 0.33, 95.23 ± 1.04, 94.93 ± 1.00 and 90.43 ± 2.28, respectively.


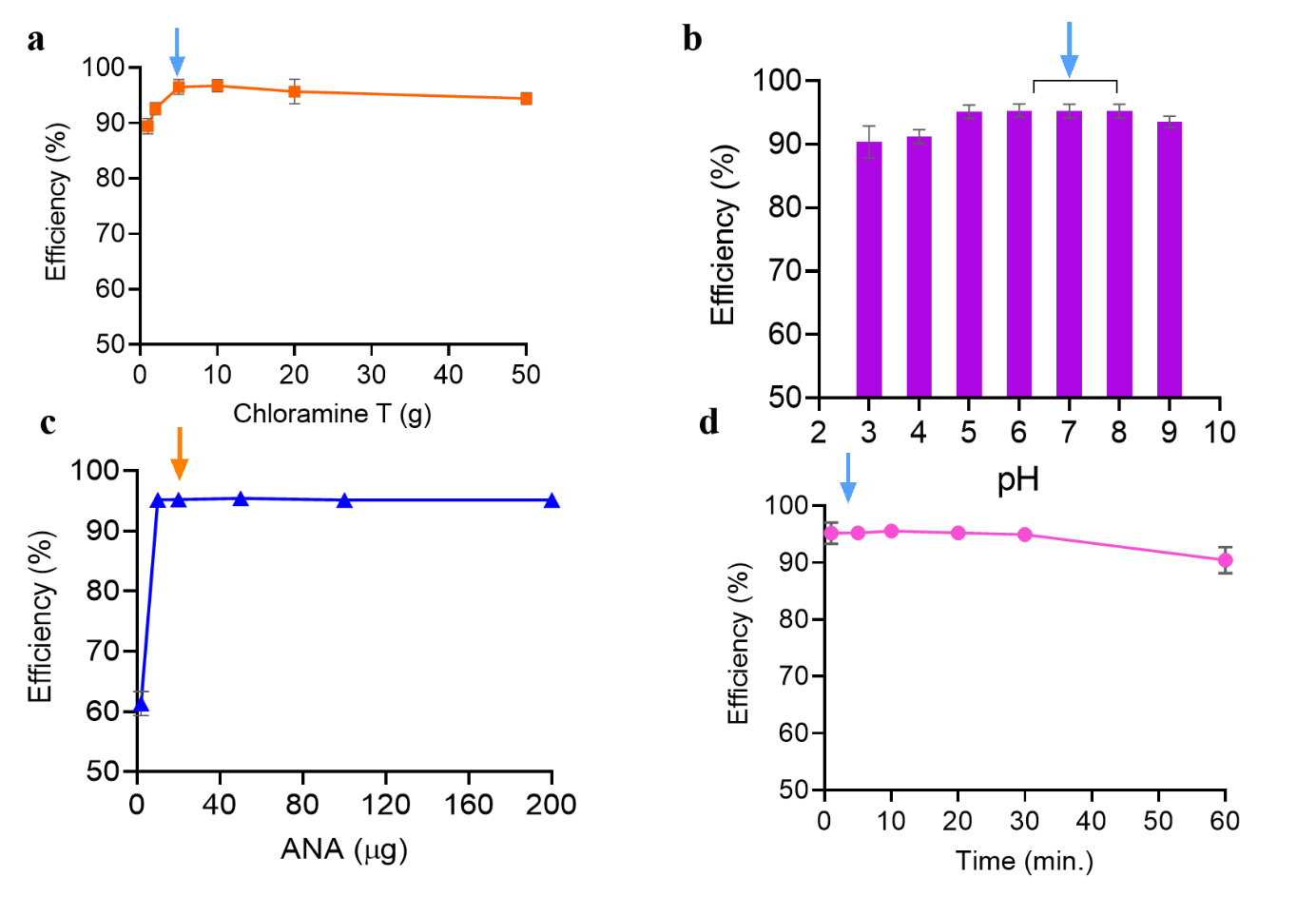


**FIGURE S1**. Optimization of radiolabeling conditions for ^131^I-ANAs preparation. (**a**) effect of Chloramine T quantity, (**b**) Effect of pH. (**c**) Effect of ANA content, (**d**) Effect of incubation time. The Labeling efficiency is analyzed by paper electrophoresis in 0.025 M phosphate buffer, pH 7.5, 300 V, 60 minutes. Results were calculated on a Cyclone radiograph and processed with OptiQuant 5.0 software.

**Supplementary conclusions S1:** The results show that the optimal labeling efficiency for the preparation of ^9131^I-ANAs is achieved at > 95% with the chloramine T content of 5-10 μg, pH in the range 6-8, ANAs 100 - 200 μg (mole ratios of ^131^I to ANA are 2.6:1 - 1.3:1) and incubation time was 5-10 minutes.

**2) Supplementary method S2:** In the treatment experiment, 50 BALB/c mice with PC3 xenografts were divided into 3 groups and given six doses of weekly injections with 100 μl 0.9% NaCl (n=10), 20 μg ANAs (n=10) or ~7.33 MBq/20 μg ^131^I-ANAs (n=30). The body weight was measured every 3-4 days. The differences in weight was calculated using Graphpad 8.

**Supplementary results S2:** After six administered doses of 0.9% NaCl, ANAs and ^131^I-ANA, nude mice exhibited no significant decrease in body weight compared to mice treated with saline control (P > 0.05, two-way ANOVA; **Fig. S2**). The initial body weight for the 0.9% NaCl, ANAs and ^131^I-ANA group were 24.70 ± 1.03 g, 23.88 ± 1.09 g and 25.94 ± 0.79 g (mean ± SEM), respectively. On Day 21, the average body weight for 3 groups were 23.28 ± 1.85 g, 24.8 ± 1.81 g and 23.22 ± 0.82 g, respectively. On Day 45, the body weight for 3 groups were 21.28 ± 1.33 g, 24.44 ± 1.84 g and 22.60 ± 0.67 g. The mean difference in weight on Day 1 for the 3 groups were insignificant (NaCl vs. ANA: 0.81 g, P = 0.85; NaCl vs. ^131^I-ANAs: -1.24 g, P=0.71; ^131^I-ANAs vs. ANA: -2.05 g, P=0.51, two-way ANOVA with Tukey's multiple comparisons test). Similarly, the mean difference in weight between the 3 groups were insignificant on Day 21 (NaCl vs. ANA: -1.51 g, P=0.51; NaCl vs. ^131^I-ANAs: 0.0 6g, P=0.99; ^131^I-ANAs vs. ANA: 1.57 g, P=0.54) and Day 45 (NaCl vs. ANA: -3.16 g, P=0.22; NaCl vs. ^131^I-ANAs: -1.320 g, P=0.605; ^131^I-ANAs vs. ANA: 1.84g, P=0.62).


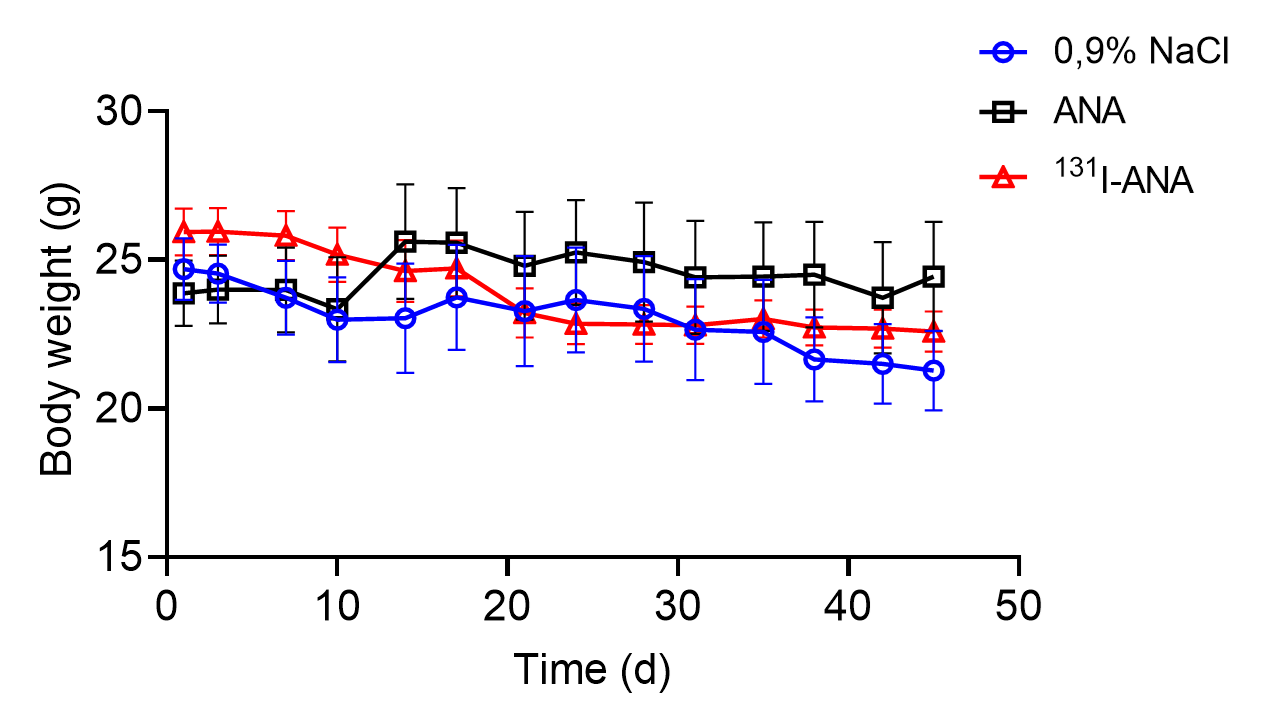


**FIGURE S2.** Body weight in nude mice bearing PC3 tumor xenografts (mean ± SEM, n=10 in the 0.9% NaCl and ANA groups, n=30 in the ^131^I-ANA group). There were no significant differences in weight with Row Factor (P = 0.531) and Column Factor (P = 0.057), two-way ANOVA.

**Supplementary conclusions S2:** The results show that no significant differences in body weights were seen in the experiments mice after injection with multiple doses of either 0.9% NaCl, ANAs and ^131^I-ANA for 45 days.
